# Intestinal Spirochetosis and Chronic Diarrhea: A Case Report and Literature Review

**DOI:** 10.7759/cureus.40276

**Published:** 2023-06-11

**Authors:** Samuel D Novick, Mefthe Berhanu, Yordanos G Negassi, Selamawit W Demissie, Syeda Areeba Hussain Kazmi, Shaniah S Holder

**Affiliations:** 1 General Surgery, Nassau University Medical Center, East Meadow, USA; 2 Medical School, University of Nicosia Medical School, Nicosia, CYP; 3 Health Science, University of Texas Health Science Center at Houston, Houston, USA; 4 Internal Medicine, Learn and Live Wholestic Health Services Clinic, Alexandria, USA; 5 Internal Medicine, Global Health Services, Virginia, USA; 6 Internal Medicine, Karachi Medical and Dental College, Karachi, PAK; 7 Medicine, American University of Barbados School of Medicine, Bridgetown, BRB

**Keywords:** histopathology, non-immunodeficient, rectosigmoid biopsy, chronic watery diarrhea, spirochetosis

## Abstract

Spirochetosis is a rare condition characterized by the presence of spirochetes in the gastrointestinal tract. It is typically associated with immunodeficiency. We present a case of chronic watery diarrhea in a 48-year-old housewife who had a 12-week history of variable-volume bowel movements without blood or mucus, accompanied by a sense of urgency. Chronic diarrhea led to weight loss and fatigue, significantly impacting her quality of life. Despite the absence of known risk factors, a comprehensive clinical evaluation and exclusion of other potential causes prompted a rectosigmoid biopsy, which revealed distinctive histological findings of spirochetosis. This case underscores the significance of considering spirochetosis as a differential diagnosis in cases of chronic watery diarrhea, even in the absence of immunodeficiency. The utilization of rectosigmoid biopsy and careful histopathological examination played a pivotal role in establishing an accurate diagnosis.

## Introduction

Diarrhea is defined as a change in stool consistency corresponding with types five to seven on the Bristol stool chart in addition to an increased stool frequency of greater than three stools daily [1]. If these symptoms last longer than four weeks, it is known as chronic diarrhea [2]. The differential diagnoses are broad and include (i) irritable bowel syndrome (IBS), (ii) inflammatory bowel disease such as Crohn’s disease, (iii) microscopic colitis, (iv) systemic disorders such as hyperthyroidism, (v) medications including antibiotics, antacids, and chemotherapeutic agents, and (vi) malabsorptive syndromes such as celiac disease, chronic pancreatitis, and lactose intolerance [2]. Chronic infections of the gastrointestinal tract have been linked to diarrhea. Common microbial culprits include *Clostridium difficile*, *Vibrio cholerae*, *Salmonella*, *Shigella*, and *Giardia* [2]. However, in rare cases, spirochete colonization in the colon may lead to chronic diarrhea in addition to other symptoms, this is known as human intestinal spirochetosis (IS).

Human IS is a rare condition that is characterized by the presence of spirochetes within the apical membrane of the colorectal epithelium of the large intestines [3]. The most common microorganisms that cause IS include the anaerobic bacteria *Brachyspira pilosicoli*,which is an opportunistic pathogen, and *Brachyspira aalborgi*, which is a non-pathogenic commensal that is part of the intestinal tract of humans and animals [4]. The prevalence of IS is not well documented, but higher rates of spirochete colonization and subsequent infection ranging from 10.8% to 64.8% are found in developing countries in Asia, such as India and Indonesia, Australia, and Africa [4,5]. Over the years, the reported rates of IS in the Western world have been on the rise with an estimated prevalence of 1-5% [6]. A study in Chicago in the 1900s discovered intestinal spirochetes in the feces of 28% of healthy persons tested [3].

Habitation in unhygienic conditions with exposure to contaminated water and infected animals continues to be the most significant risk factor for IS in poorly developed areas [6]. In developed countries, immunocompromised states such as infection with human immunodeficiency virus (HIV) and persons who engage in homosexual intercourse, specifically men who have sex with men (MSM), are common risk factors and are associated with the highest prevalence with rates ranging between 20.6% and 62.5% [3,7]. This leads to the question of whether IS in the developed world can be classified as a sexually transmitted infection (STI) [8]. Although the risk is higher in these individuals, there are some cases of IS occurring in heterosexual and immunocompetent persons [9]. The presence of spirochetes in the colon or stool may not lead to the clinical manifestations of IS and symptoms of this condition do not reveal the exact location of the microorganisms in the intestine [6]. Colonization may occur in any portion of the colon with reported cases of isolated involvement of the rectum or proximal colon only or within the whole length of the large intestine [5]. This contributes to the heterogeneous nature of the symptomatic presentation of IS [5]. The severity of IS symptoms is broad and ranges from asymptomatic colonization to severe features, including abdominal pain, chronic diarrhea, meteorism, nausea and vomiting, hematochezia, and weight loss [10]. Severe cases of IS in critically ill patients may lead to spirochetemia, sepsis, and multiple organ failure [3].

In this report, we present the case of a previously healthy, 48-year-old female with a 12-week history of chronic diarrhea who was subsequently diagnosed with IS via rectosigmoid biopsy.

## Case presentation

A 48-year-old African housewife, previously healthy, presented to our gastroenterology clinic with a chief complaint of chronic watery diarrhea and vague abdominal pain persisting for the past 12 weeks. She complained of multiple loose and watery bowel movements per day, accompanied by a sense of urgency and abdominal pain. The volume of each bowel movement was variable, ranging from small to moderate. There were no reports of blood or mucus in the stool. The patient described a sense of urgency associated with bowel movements, and there were no specific triggers or exacerbating factors identified. The chronic watery diarrhea had significantly impacted the patient’s daily life, resulting in weight loss (2-3 kg) and fatigue. The patient reported no recent history of travel and denies the use of any recent antibiotics or medications. However, in an attempt to alleviate her diarrhea, she resorted to over-the-counter (OTC) medications (bismuth and loperamide), but, unfortunately, there was no improvement in her condition. She was married and reported no history of multiple sexual partners. However, she admitted to engaging in anal intercourse with her husband in the past.

On physical examination, the patient appeared malnourished, and mild tenderness was elicited on abdominal palpation without any palpable masses or organomegaly. Vital signs were as follows: blood pressure was 100/70 mmHg, and heart rate was increased to 110 beats per minute with a normal body temperature. Stool samples were collected for microbiological analysis, including testing for bacterial, viral, and parasitic pathogens, as well as fecal calprotectin levels to assess for underlying inflammation. Additionally, blood tests were performed, including a complete blood count, liver and renal function tests, serum electrolytes, and inflammatory markers. The laboratory results showed no significant abnormalities, ruling out common causes such as infectious agents, inflammatory bowel disease, and malabsorption syndromes. The patient underwent further investigations, including celiac serology, and thyroid function tests, which did not reveal any specific abnormalities. Table 1 shows all lab parameters.

**Table 1:** Lab parameters of the patient.

Laboratory test	Parameter	Result	Normal range
Complete blood count	Hemoglobin	13.6 g/dL	12.1–15.1 g/dL
	Hematocrit	46 %	36–48%
	White blood cell count	11.1 cells/mm³	4.5–11 cells/mm³
	Platelet count	336 cells/mm³	150–400 cells/mm³
Liver function tests	Alanine aminotransferase (ALT)	23 U/L	7–55 U/L
	Aspartate aminotransferase (AST)	31 U/L	8–48 U/L
	Alkaline phosphatase	200 U/L	44–147 U/L
	Total bilirubin	0.9 mg/dL	0.8–1.2 mg/dL
	Albumin	4.1 mg/dL	3.4–5.4 g/dL
	Total protein	68 g/dL	60–80 g/dL
Renal function tests	Blood urea nitrogen (BUN)	18 mg/dL	6–24 mg/dL
	Creatinine	1.0 mg/dL	0.7–1.3 mg/dL
	Estimated glomerular filtration rate	100 mL/min/1.73m²	>60 mL/min/1.73m²
Electrolytes	Sodium (Na)	137 mmol/L	135–145 mmol/L
	Potassium (K)	3.6 mmol/L	3.5–5.0 mmol/L
Thyroid function tests	Thyroid-stimulating hormone (TSH)	2.5 µIU/mL	0.4–4.0 µIU/mL
	Free T4	1.6 ng/dL	0.9–2.3 ng/dL
Inflammatory markers	C-reactive protein (CRP)	14 mg/L	<10 mg/L
	Erythrocyte sedimentation rate	30 mm/hour	0-20 mm/hour
Fecal calprotectin	Fecal calprotectin level	20 µg/mg	10–60 µg/mg
Stool microbiological analysis	Bacterial pathogens	Negative	
	Viral pathogens	Negative	
	Parasitic pathogens	Negative	

Considering the patient’s sexual history, which involved anal intercourse with her husband, additional investigations were pursued to explore the possibility of an STI contributing to her symptoms. Specific laboratory tests were performed to screen for common STIs, including gonorrhea, chlamydia, syphilis, and HIV. However, these tests did not provide evidence of an STI.

**Figure 1:**
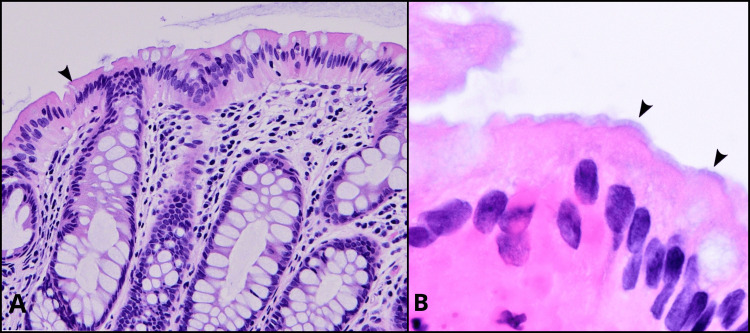
Histological sections of the biopsy specimen showing spirochetosis using hematoxylin and eosin staining (arrowhead). A: 10× zoom. B: 40× zoom.

Despite the extensive evaluation, the chronic watery diarrhea persisted, and the patient’s quality of life continued to decline. To further investigate the underlying cause, a rectosigmoid biopsy was performed. The rectosigmoid biopsy specimens were subjected to histopathological examination. Microscopic analysis (light microscope) revealed the presence of spirochetes within the mucosal lining of the rectosigmoid colon. The histological analysis of spirochetosis demonstrated a notable, blurred blue line at the luminal border of the colonic mucosa upon examination with hematoxylin and eosin (H&E) staining. Additionally, the findings revealed the presence of non-specific inflammation characterized by lymphocytic, neutrophilic, or eosinophilic infiltration within the lamina propria or epithelium (Figure 1).

**Figure 2:**
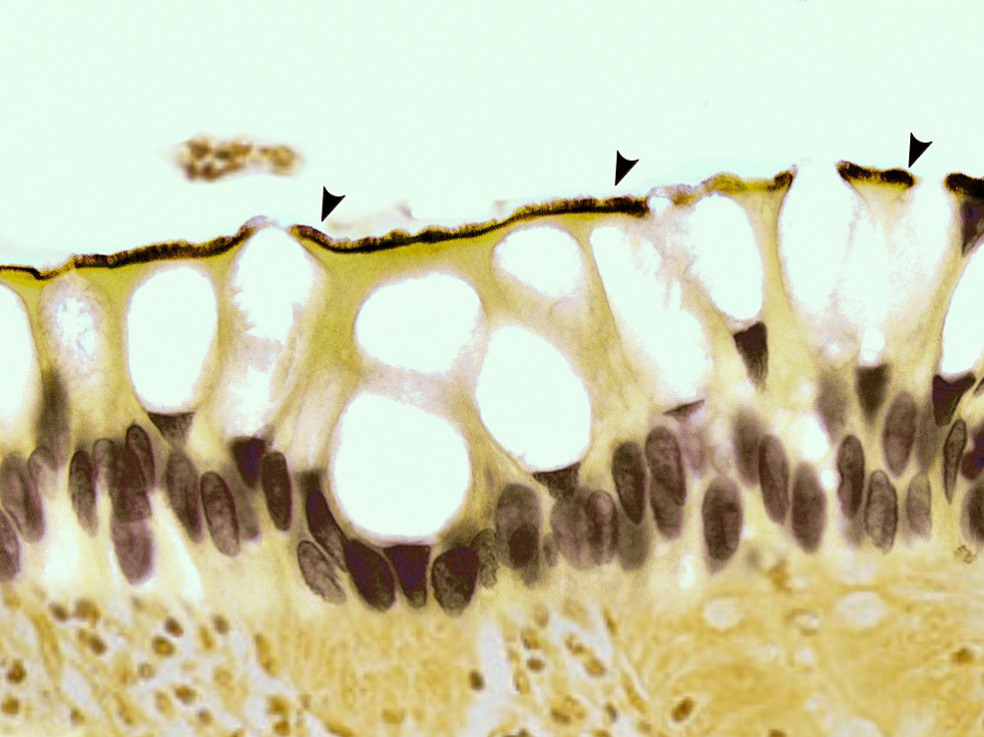
Histological section of the biopsy specimen showing spirochetosis using Warthin-Starry silver staining (arrowhead).

A special silver stain, Warthin-Starry silver, was used to highlight the spirochetes, confirming the diagnosis of spirochetes (Figure 2).

The presence of characteristic spiral-shaped organisms within the colon mucosa was consistent with gastrointestinal spirochetosis. Notably, the identified spirochetes were consistent with *B. aalborgi* infection.

To effectively manage the patient’s condition, a course of metronidazole 500 mg four times daily for a duration of two weeks was initiated. Following the commencement of treatment, the patient reported a significant improvement in her symptoms, with a reduction in the frequency of bowel movements and an improvement in stool consistency. The patient was closely monitored during the treatment period, and no adverse effects were reported. The patient’s energy levels improved, and she regained the weight she had lost during the illness.

## Discussion

*B. aalborgi* and *B. pilosicoli* belong to the Brachyspiraceae family, which is one of the three families of spirochetes [9]. The other two are Spirochaetaceae, comprising *Borrelia*, *Spironema*, *Spirochaeta*, and *Treponema*, and Leptospiraceae including the bacteria *Leptonema *and *Leptospira *[9]. Infections caused by bacteria from the Leptospiraceae and Spirochaetaceae families may also cause diarrhea and are more common, making the diagnosis of IS more difficult. For example, infection with *Treponema pallidum* may cause syphilitic proctitis, which is rare, and presents with diarrhea and lower abdominal pain with rectal biopsy histological findings of non-specific inflammation and spirochetes on silver stain [11]. Therefore, due to some overlapping symptoms, extensive patient history, lab work, and physical examination are required to differentiate between IS bacteria and other spirochetes.

The exact pathogenesis of IS and the mode of transmission in immunocompetent persons is unknown; however, some studies suggest that the bacteria can be transmitted via a fecal-oral route from contaminated water or poultry which may be the primary sources of infection in developing countries [9]. MSM regardless of HIV status have an increased risk of being colonized with *B. pilosicoli* and are more likely to be symptomatic when compared to IS in heterosexual persons which is commonly caused by *B. aalborgi* [12]. Studies have found that in MSM, some cases of IS are associated with gonococcal and/or *Shigella flexneri* coinfection indicating that a sexual route of transmission is possible [8]. It is theorized that the oro-anal sexual practices of MSM may alter the rectal microenvironment increasing the risk of colonization and infection [12]. Although patients with HIV and IS tend to be symptomatic, there appears to be no correlation between the degree of immunodeficiency and the extent of the disease [3].

These bacteria are slow growing with estimated growth times of six days (*B. pilosicoli*) and two weeks (*B. aalborgi*), and because intestinal colonization is asymptomatic, the duration of the incubation period is unknown [3]. Due to the heterogeneous nature of IS, the clinical presentation varies. The most common symptom is chronic diarrhea which is usually watery in character [10]. These opportunistic anaerobes have a cytopathic effect by destroying the microvilli on the absorptive epithelial cells and inducing changes in the cellular cytoskeleton [8]. This may lead to chronic secretory diarrhea with associated symptoms of abdominal pain, nausea, vomiting, and weight loss due to nutrient loss [8]. In some cases, colonic invasion and inflammation may lead to hematochezia [7].

Diagnosis begins with a thorough patient history and laboratory tests to rule out the differential diagnoses of chronic diarrhea. Due to the rare nature of this condition, a high index of clinical suspicion is required to establish the diagnosis and begin appropriate treatment. An endoscopic view of the colon via colonoscopy commonly yields normal results with occasional cases of erythematous areas present [7]. A study by Alsaigh and Fogt evaluated the various clinical presentations of 15 patients with a confirmed diagnosis of IS [13]. Colonoscopy findings were described as normal in six patients, polyoid in seven patients, erythematous in one patient, and a lesion in one case [13]. This indicates that normal colonoscopy results are not sufficient to rule out the diagnosis of IS. The histological view of a biopsy sample is the gold standard diagnostic test, and this disease is characterized by the appearance of a 3-6 μm diffuse blue fringe along the intercryptal epithelial layer on the H&E stain [10]. Visualization of the spirochetes on Warthin-Starry or other silver stains confirms the diagnosis [10]. In some cases, organisms may be non-invasive and found on the cell surface; however, intraepithelial mast cell and IgE plasma cell infiltration may occur, leading to colonic inflammation [3]. Microvilli blunting, glycocalyx defects, and mitochondrial swelling may also occur [10]. The degree of cellular destruction positively correlates with the degree of spirochete invasion and severity of symptoms, with diarrhea being more pronounced in persons with severe microvilli destruction and higher spirochete attachment [3].

Colonization varies from patient to patient and may be isolated or affect the entire colon including the rectum [5]. Studies have found that intestinal spirochetes have a tendency to affect the right side and ascending colon; however, in some cases like this one, isolated rectosigmoid involvement may occur [10]. Biopsies in the ascending colon have a success rate of diagnosis of 56%, followed by the transverse colon (54%), descending colon (48%), sigmoid colon (47%), cecum (40%), and, lastly, the rectum (38%) [4]. In this case, the colonoscopy with biopsy of the ascending, transverse, and descending colon yielded normal results while the rectosigmoid biopsy aided in confirming the diagnosis. This signifies that isolated colonic biopsies may lead to missing the diagnosis, therefore, a biopsy from each segment of the colon will confirm or completely rule out IS. Research by Tanahashi et al. discovered that immunostaining with anti-*T. pallidum* and anti-*M. bovis* antibodies cross-react with spirochetes and aids in the visualization of spirochetes on the epithelial surface [14]. They are easily available and have been found to provide clear staining [14]. Newer methods of identifying IS bacteria are being explored. Fecal polymerase chain reaction (PCR) targeting the 16S ribosomal ribonucleic acid (rRNA) subunit and reduced nicotinamide adenine dinucleotide (NADH) oxidase genes specific for *B. pilosicoli* and *B. aalborgi* show promising results [15]. PCR techniques are useful for specifically detecting the presence of *B. aalborgi* or *B. pilosicoli* DNA in biopsied tissue samples; however, they do not provide information about the distribution or extension of the organisms in the sample [15].

Antimicrobial therapy, specifically metronidazole monotherapy, is the most effective treatment modality for IS [4]. Its efficiency surpasses macrolide, clindamycin, and macrolide plus metronidazole use [10]. In cases of coinfection, combination antibiotic therapy may be required. Treatment dosage and duration may differ and depend on factors such as age and weight; however, patients are normally given metronidazole for 10 days [10]. Although capable of symptom resolution, some patients treated with metronidazole have reported symptomatic relapse when following up. This may be due to incomplete eradication or re-infection from environmental sources [4]. A study by Jabbar et al. found that metronidazole treatment paradoxically promoted *Brachyspira *relocation into the colonic crypts and goblet cell granules representing a possible bacterial strategy to elude antibiotic exposure [16]. This may also be a possible cause of symptom continuance and relapse; therefore, further research on spirochete antibiotic sensitivity and alternative modes of treatment is required for more effective management. Some cases of IS have been reported to spontaneously resolve [10].

IS has been reported in the literature with new discoveries surfacing every few years. Table 2 summarizes various reports highlighting the patients’ presentations, significant diagnostic findings, and the treatment and prognosis [8-10,13,14,17-20]. In addition to the cases mentioned, our case aims to increase physician awareness about the heterogeneous nature of intestinal spirochetosis and the importance of considering it as a differential diagnosis in patients presenting with chronic diarrhea.

**Table 2:** The patients’ presentations, significant diagnostic findings, and the treatment and prognosis of the cases reported in the literature. IS: intestinal spirochetosis; N/S: not specified, n: number of persons affected; PCR: polymerase chain reaction, B.A: *Brachyspira aalborgi*;  B.P: *Brachyspira pilosicoli*; PB: penicillin benzathine; STD: sexually transmitted disease; U/S: ultrasound; ESR: erythrocyte sedimentation rate; CRP: C-reactive protein

Author, year	Country	Patient sample	Risk factors	Symptoms	Significant colonoscopy findings	Additional biopsy findings	Additional tests	Management and prognosis
Esteve et al. 2006 [17]	Spain	11 positive cases out of 1,174 samples	N/S	Chronic watery diarrhea (n = 8)	Unspecified suspicious findings (n = 3)	Classic findings of IS in 11 patients (8 with normal colonoscopy and 3 with abnormal colonoscopy)	PCR amplification in 2 samples found B.A (n = 1) and B.P (n = 1)	Metronidazole with clinical and histological resolution (n = 3). PB with clinical and histological resolution (n = 3)
Tanahashi et al. 2007 [14]	Japan	20 positive cases out of 2,556 samples with an age range of 35 to 75 years	None except one case of HIV	Lower abdominal pain, melena, and diarrhea in 9 patients	Varied, but most specimens were obtained from polypoid lesions	20 positive cases with characteristic findings. Inflammatory reaction was slight in most cases. The HIV-positive case was accompanied by erosion and marked inflammation	PCR amplification + in 20 samples. B.A was detected in all cases, 3 of which revealed dual infection of both species	Unspecified antibiotic therapy for symptomatic patients
Garcia-Hernandez et al. 2021 [8]	Barcelona	6 positive cases of IS with a median age of 31.5 years	MSM (n = 6). HIV (n = 3)	Diarrhea (n = 5)	No significant findings	All cases with classic features. Mild inflammatory changes were found in 5 cases	STD testing found concomitant rectal gonorrhea in 2 patients	Metronidazole in 5 patients and PB in 1 patient with recovery in all
Lemmens et al. 2017 [10]	Belgium	4-year-old male	Recent infection with C. Jejuni treated with antibiotics	Abdominal pain and hematochezia	Rectal irritation	Classic findings of IS with mild lymphocytic infiltration noted	None	Amoxicillin-clavulanic acid with a resolution of symptoms
Weisheit et al. 2007 [18]	Scandinavia	209 patients with confirmed IS, 168 (80.4%) were males, with a mean age of 50.75 years	MSM (6.5%) HIV (3.8%)	Abdominal pain (46%), diarrhea (51%), and alternating diarrhea and constipation (13%)	N/S	Classic findings of IS only	None	72/84 patients received metronidazole, and the symptoms improved in 44 of the 84 patients. Biopsies in 20 of these patients no longer revealed infection with spirochetes, and symptoms were found to have improved in 11 of the 20 patients
Alsaigh et al. 2002 [13]	United States	15 (11 males, 4 females) patients with colonic biopsies that showed spirochetosis	MSM (n = 1)	Chronic watery diarrhea (40%). Change in bowel habits (33%).	Normal (n = 6). Polyoid (n = 7). Erythematous (n = 1). Lesion (n = 1)	Classic findings of IS only in all patients	None	N/S
Lemmens et al. 2017 [10]	Belgium	8-year-old male	A recent vacation to an endemic region	Abdominal pain, chronic diarrhea, and perianal irritation	No significant findings	Classic findings of IS only	None	The first course of metronidazole for 2 weeks with relapse at 15 months and 2 years after initial treatment. The patient was treated on both occasions with a course of metronidazole. Complete resolution of symptoms after the second relapse
Heine et al. 2008 [19]	Australia	4 children and adolescents (2 males and 2 females with an age range of 9–16 years	None	Persistent diarrhea (n = 2). Rectal bleeding (n = 1). Abdominal pain (n = 2)	N/S	Classic findings of IS only	PCR amplification found B.A in all 4 patients	Unspecified antibiotic therapy
Surawicz et al. 1987 [20]	N/S	130 males	MSM (n = 130)	Intestinal symptoms (92%)	No significant findings	Classic findings on rectal biopsy (n = 39)	Rectal gonorrhea and unspecified intestinal pathogens found in some cases	N/S
Lemmens et al. 2017 [10]	Belgium	7-year-old boy	None	Bloody diarrhea, abdominal pain, perianal rash, encopresis	Mild rectal irritation	Rectosigmoid biopsy showed classic findings of IS	Stool culture - negative. Abdominal U/S - negative. Serology - negative	Metronidazole for 10 days with resolution of abdominal pain and bloody diarrhea but perianal rash and encopresis continued
Perez Moux et al. 2022 [7]	N/S	57-year-old male	MSM HIV	4-month history of intermittent bloating and frequent hematochezia	A few small, 2-3 mm, superficial, erythematous lesions on the ascending colon	Classic findings of IS only	Serology - negative	Metronidazole 500 mg 4 times a day for 10 days with complete resolution of symptoms
Lemmens et al. 2017 [10]	Belgium	12-year-old boy	None	Abdominal pain, non-bloody diarrhea, and loss of appetite	No significant findings	Classic findings with mild signs of inflammation in the colonic mucosa and cryptitis in the cecum and transverse colon	Stool culture + for Salmonella. Serology - elevated CRP (16.6 mg/L)\. Elevated ESR (16 mm/h). Abdominal U/S - mesenteric adenitis and colitis	Metronidazole and amoxicillin with complete resolution of symptoms
Guzman Rojas et al. 2018 [9]	N/S	66-year-old male	None	Asymptomatic	Colitis in the cecum and at the ileocecal valve	Classic findings of IS with mild chronic non-specific inflammation in the cecum	None	No treatment since asymptomatic

This patient had a 12-week history of voluminous watery diarrhea due to intestinal spirochetosis. Although she was immunocompetent, her sexual practices may have been a risk that contributed to her condition. Extensive diagnostic tests with stool assay, serology, and colonoscopy all yielded negative results; however, rectosigmoid biopsy with the use of Warthin-Starry silver stain aided in confirming the diagnosis of IS. Monotherapy with metronidazole led to the complete symptomatic resolution and aided in returning this patient to her previous level of functionality.

## Conclusions

Human IS is a rare gastrointestinal condition that remains poorly understood. IS manifests with a wide range of symptoms, with chronic diarrhea being the most common presentation. Given the extensive list of potential causes for chronic watery diarrhea, IS often goes unnoticed and undiagnosed in many individuals. This case underscores the importance of considering spirochetosis as a potential differential diagnosis in cases of chronic watery diarrhea, even in individuals without immunodeficiency or predisposing factors. Therefore, maintaining a high index of clinical suspicion and employing appropriate diagnostic measures, such as colonic biopsy with Warthin-Starry silver staining, along with targeted treatment using metronidazole for symptomatic patients, are crucial.

While spirochetes typically colonize the ascending colon, there have been instances of isolated rectal involvement, underscoring the importance of conducting rectosigmoid biopsies to effectively rule out the diagnosis. Additionally, PCR testing for *B. aalborgi* and *B. pilosicoli*, as well as anti-treponemal and anti-*M. bovis* antibody testing, has shown promise in aiding the diagnosis of IS. Physicians should be aware of this condition and its diverse range of symptoms, particularly in endemic areas, to facilitate timely management and enhance patients’ quality of life.
